# Physiological, Biochemical and Molecular Assessment of UV-A and UV-B Supplementation in *Solanum lycopersicum*

**DOI:** 10.3390/plants10050918

**Published:** 2021-05-03

**Authors:** Nuno Mariz-Ponte, Rafael J. Mendes, Sara Sario, Cristiana V. Correia, Carlos M. Correia, José Moutinho-Pereira, Paula Melo, Maria Celeste Dias, Conceição Santos

**Affiliations:** 1 Department of Biology, Faculty of Sciences, University of Porto, Rua do Campo Alegre, 4169-007 Porto, Portugal; rafael.mendes@fc.up.pt (R.J.M.); sara.sario@fc.up.pt (S.S.); cristiana.correia@fc.up.pt (C.V.C.); pmmelo@fc.up.pt (P.M.); csantos@fc.up.pt (C.S.); 2 LAQV-REQUIMTE, Faculty of Science, University of Porto, Rua do Campo Alegre, 4169-007 Porto, Portugal; 3 Centre for the Research and Technology of Agro-Environmental and Biological Sciences (CITAB), Department of Biology and Environment, University of Trás-os-Montes e Alto Douro, 5001-801 Vila Real, Portugal; ccorreia@utad.pt (C.M.C.); moutinho@utad.pt (J.M.-P.); 4 Centre for Functional Ecology, Department of Life Sciences, University of Coimbra, Calçada Martim de Freitas, 3000-456 Coimbra, Portugal; celeste.dias@uc.pt

**Keywords:** fruiting stage, indoor growth, photosynthesis, tomato, ultraviolet supplementation

## Abstract

Daily UV-supplementation during the plant fruiting stage of tomato (*Solanum lycopersicum* L.) growing indoors may produce fruits with higher nutraceutical value and better acceptance by consumers. However, it is important to ensure that the plant’s performance during this stage is not compromised by the UV supplement. We studied the impact of UV-A (1 and 4 h) and UV-B (2 and 5 min) on the photosynthesis of greenhouse-grown tomato plants during the fruiting/ripening stage. After 30 d of daily irradiation, UV-B and UV-A differently interfered with the photosynthesis. UV-B induced few leaf-necrotic spots, and effects are more evidenced in the stimulation of photosynthetic/protective pigments, meaning a structural effect at the Light-Harvesting Complex. UV-A stimulated flowering/fruiting, paralleled with no visible leaf damages, and the impact on photosynthesis was mostly related to functional changes, in a dose-dependent manner. Both UV-A doses decreased the maximum quantum efficiency of photosystem II (Fv/Fm), the effective efficiency of photosystem II (ΦPSII), and gas exchange processes, including net carbon assimilation (P_N_). Transcripts related to Photosystem II (PSII) and RuBisCO were highly stimulated by UV supplementation (mostly UV-A), but the maintenance of the RuBisCO protein levels indicates that some protein is also degraded. Our data suggest that plants supplemented with UV-A activate adaptative mechanisms (including increased transcription of PSII peptides and RuBisCO), and any negative impacts on photosynthesis do not compromise the final carbohydrate balances and plant yield, thus becoming a profitable tool to improve precision agriculture.

## 1. Introduction

Tomato (*Solanum lycopersicum* L.) is amongst the most popular and consumed crop species worldwide, with vast agricultural and economic importance. Around 182.3 million tons of tomato fruits were produced in 2018 (www.fao.org/faostat/, accessed in September of 2020), generating a global revenue of 190.4 billion dollars [1]. Its excellent acceptance by the consumers is due to its multiple gastronomic uses and other features like its taste, color, and high nutritional value [2,3].

This crop is produced in open fields and indoors, including greenhouses [4]. Tomato production outdoors, which is widely used in the pulp industry, allows plants to grow so that they are naturally adapted to the solar ultraviolet (UV) radiation. In indoor cultures (mostly dedicated to table tomato), UV-exposure is usually neglected, since greenhouse building materials are diverse and include glass and polycarbonate, which totally or partially filter solar UV radiation. Additionally, artificial light may be used as a supplement or as the only source of light [5]. Although previous studies demonstrated that fruits and vegetables can be grown indoors out of season, they are described as having low nutritional and organoleptic attractiveness [6]. 

Nevertheless, in the last decade, a new agricultural paradigm emerged, which considers UV-supplementation as a strategy to improve crop yield and/or quality [7,8,9,10,11]. Besides, UV-irradiation systems do not pose legal restrictions, and some systems (e.g., UV-A, LED-UV) are easily affordable. Controlled UV-supplementation can be a powerful tool not only to control indoors-crop pests and diseases but also to promote the synthesis of defense compounds and increase the nutritional quality and organoleptic attributes of the edible parts, which can advance the current agriculture requirements [11,12,13]. However, it is also necessary to ensure that the application of these UV supplements does not compromise the plant’s performance (namely photosynthesis) during the fruiting stage. 

For instance, plants exposed to excessive UV rays, especially UV-B, suffer biological changes, which include decreased growth and yield, and leaf chlorosis and/or necrosis [14,15]. Defense mechanisms in response to harmful light (intensity or quality) develop defense mechanisms mediated by molecular photoreceptors [13,16,17]. Photoreceptors sense and transduce light signals through distinct intracellular signaling pathways (which include the modulation of light-regulated genes) and ultimately lead to adaptive changes at the cellular and systemic levels [18]. Effects of UV-B are usually more deleterious than a similar dose of UV-A, but high levels of UV-A may lead to responses identical to those induced by lower levels of UV-B [19]. 

During maturation, fruit tissues suffer a transition from partially to true heterotrophy, meaning dramatic metabolic changes to the plant [3]. During this stage, leaf photosynthesis is crucial to export fixed carbon (mostly sucrose) to non-photosynthetic fruit sink-tissues [20]. Thus, any change in the leaf photosynthetic efficiency during the fruiting/ripening stage will alter this “source-sink” dynamics, influencing fruit yield and quality. However, beneficial effects of small amounts of UV radiation on crop yield, and how UV-triggers photosynthetic signals that may be used in precision agriculture/horticulture are far less studied, being the majority of available studies on the UV impacts on photosynthesis, and at the same time not using the crop’s fruiting/ripening stage, thus mainly focused on the negative effects of excessive UV rather than on physiologically tolerable UV-doses [21,22,23,24,25]. The beneficial effects of small amounts of UV radiation on crop yield, and how UV-triggers photosynthetic signals that may be used in precision agriculture/horticulture are far less studied. 

UV-A light that ranges between 315 and 400 nm has beneficial effects on the growth and vigor of seedlings [10]. It also increases chlorophyll and carotenoid levels and stimulates polyphenol pathways and levels [26,27], thus it may be used to improve the yield and/or quality of crops. In this line, Lee et al. [15] used LED UV-A to increase the biomass of kale plants. We have shown that UV-A irradiation during the pre-harvest period is effective in increasing ripening synchronization and the fruit’s nutritional properties, potentially making these fruits more likable to buyers [11].

The way moderate doses of UV-rays (quality and quantity) modulate specific targets of the leaf photosynthesis (including the thylakoidal electron transport chain and the Calvin cycle) during the fruiting/ripening stage remains a matter of debate. After selecting the ideal type, dose, and period of UV supplementation to enhance the nutraceutical quality of fruits and vegetables (reviewed by Huché-Thélier et al. [28]), we must evaluate its impact on the leaf photosynthesis, including at the transcriptional and post-transcriptional levels. 

Moreover, the plant’s responses to UV are an integration of its cross-talks with both environmental factors and the developmental stage of the plant (reviewed by Yadav et al. [29]). UV-B inhibited RuBisCO activity [21], but Gao et al. [24] showed that a low dose of UV-B upregulated proteins related to chloroplast structure, light reactions, oxygen-evolving enhancer proteins, and ATP synthase. The physiological understanding of the effects to low doses of UV-A/B [25,30,31], namely regarding the photosynthetic performance and carbon metabolism, is essential to evaluate the possible application of these irradiations in indoors crop production.

Following our previous work that showed biochemical and organoleptic benefits of UV supplementation to tomato fruit [11], we aim to unveil how the different quality and intensity of low (physiologically tolerated) doses of UV-A and UV-B influence the plant photosynthesis and carbon metabolism during the ripening stage of the plants, referring to physiological, biochemical and molecular tools. By distinguishing characteristic effects associated with specific UV wavelengths, and dose, we may be able to select only precise UV-supplementation indoors, namely in greenhouses, for precision horticulture.

## 2. Results

### 2.1. Plant Growth 

Thirty days (d) after the beginning of the UV treatments, the shoot length of control plants reached 20.6 ± 2.4 cm. UV treatments significantly decreased plant length (*p* < 0.05) in UV-B 2 min conditions, where its decrease reached around 20% compared with the control (Table 1). In control and UV-A treatments, plants showed healthy leaves, with no visual detrimental effects (e.g., necrosis, chlorosis). UV-B plants were also healthy, and only occasionally showed small necrotic spots. For UV-A 4 h and UV-B 2 min conditions, a significant decrease (*p* < 0.01) in the water content % (WC) was registered (Table 1). The dry matter (DM) showed a tendency to increase with UV treatments, with significant differences for UV-A 1 h (*p* < 0.05) and UV-B 5 min (*p* < 0.01) (Table 1). Regarding flowering and fruiting, while there were no changes in the total number of flowers, there was an increase in the flower and fruit number in plants exposed to both UV-A and UV-B (statistically significant (*p* < 0.05) for UV-A 1 h and UV-B 2 min).

### 2.2. Chlorophyll a Fluorescence and Pigments

**Figure 1:**
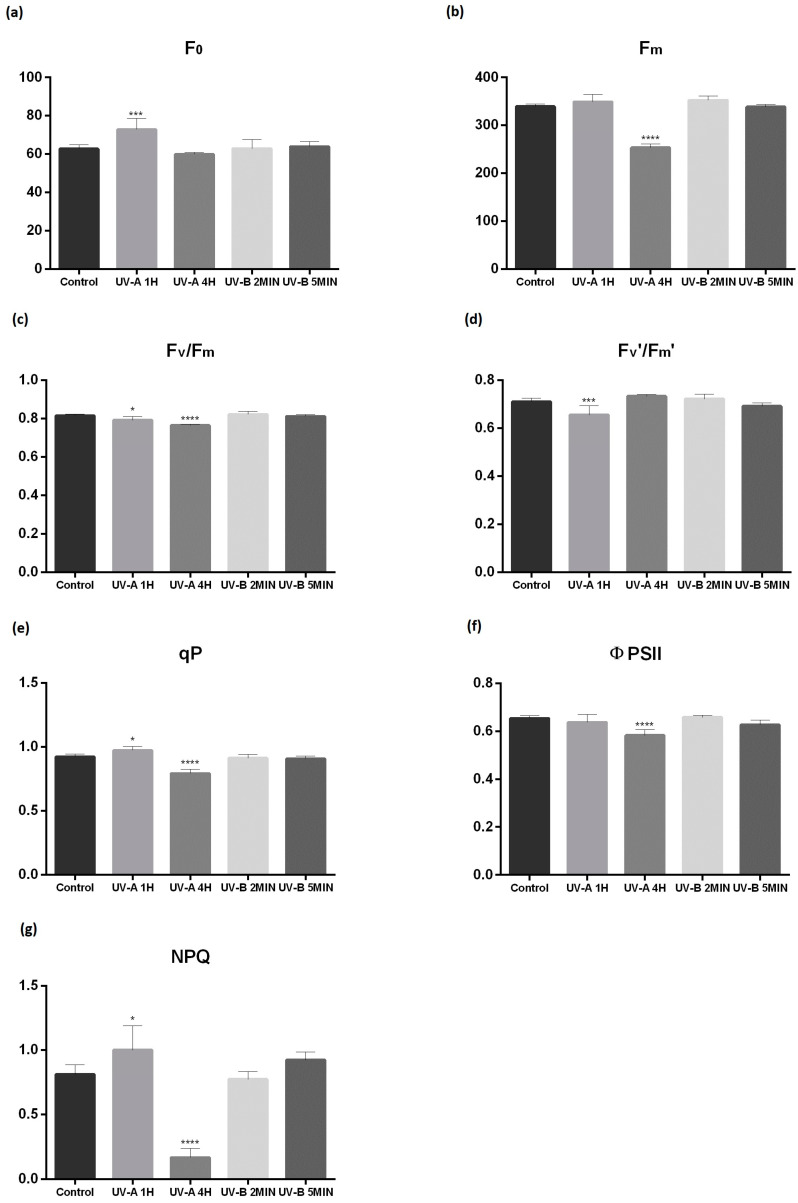
Chlorophyll *a* fluorescence after 30 d of exposure to different UV-A/B treatments. Minimal fluorescence yield of dark-adapted leaves with all PSII centers closed, F_0_ (**a**); maximum fluorescence of dark-adapted leaves with all PSII centers closed, Fm (**b**); maximum quantum efficiency of PSII, Fv/Fm (**c**); PSII maximum efficiency in saturating light if all reaction centers are open, Fv’/Fm’ (**d**); photochemical quenching, qP (**e**); effective efficiency of PSII, ΦPSII (**f**) and non-photochemical quenching, NPQ (**g**). Within each parameter, *, *** and **** mean significant differences for *p* < 0.05, 0.01, 0.001 and 0.0001, respectively. Values are expressed as the mean and standard deviation (n = 6).

Chlorophyll *a* fluorescence parameters of dark-adapted leaves were only affected by UV-A; namely, UV-A 1 h significantly increased F_0_ (*p* < 0.001) and significantly decreased Fv/Fm (*p* < 0.05) while UV-A 4 h had a more acute effect, lowering Fm and Fv/Fm (*p* < 0.0001, Figure 1a–c). UV-B did not significantly affect chlorophyll *a* fluorescence. Similarly, the corresponding ratio Fv’/Fm’ of the light-adapted condition was also significantly affected (*p* < 0.001) but only by the low doses of UV-A (Figure 1d). Both qP and NPQ were only significantly affected by the UV-A 1 h and 4 h conditions (*p* < 0.05 and 0.0001, respectively), following an hormesis effect, where UV-A 1 h treatment led to an increase and UV-A 4 h to a reduction (Figure 1e,g). The ΦPSII significantly decreased in plants exposed to UV-A 4 h (*p* < 0.0001) (Figure 1f).

Regarding pigments, UV-B radiation was more effective in increasing the levels of photosynthetic pigments (Chl a, Chl b, and carotenoids) than UV-A, with significant differences for UV-B 5 min (*p* < 0.01). The increases in Chl a were slightly higher than those in Chl b, raising the Chl a/Chl b ratios significantly in plants exposed to UV-B 2 min (*p* < 0.05). On the other hand, anthocyanins significantly decreased in all UV conditions (*p* < 0.05, 0.01, and 0.0001) (Table 2).

### 2.3. Gas Exchange, Carbohydrates, and RuBisCO

**Figure 2:**
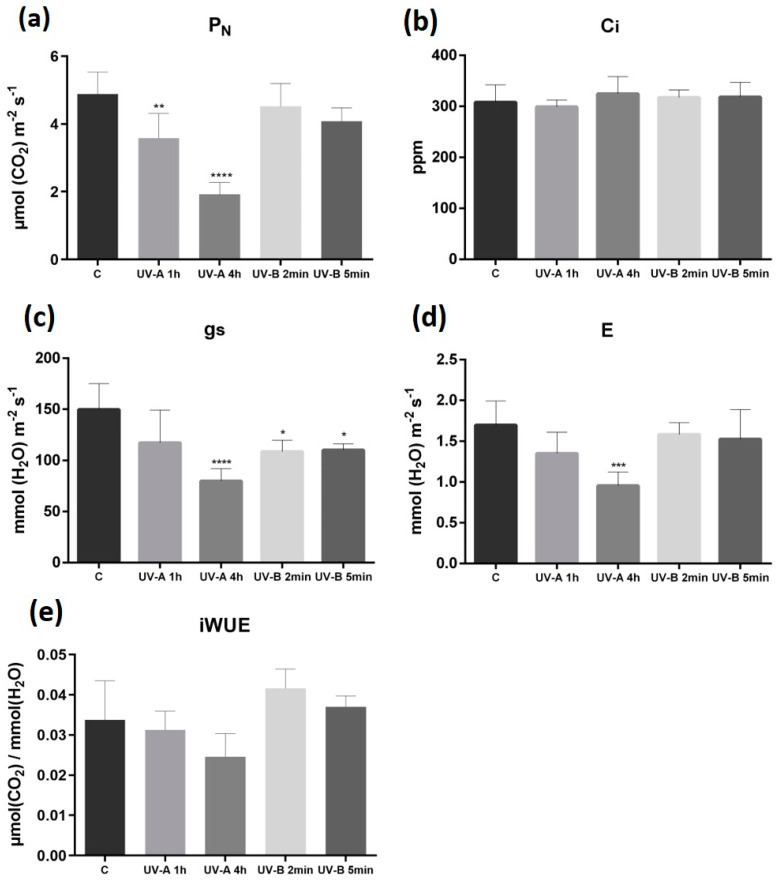
Leaf gas-exchange after 30 d of exposure to different UV-A/B conditions. Net photosynthetic rate, P_N_ (**a**); intercellular CO_2_ concentration, Ci (**b**); stomatal conductance, gs (**c**); transpiration rate, E (**d**); intrinsic water-use efficiency iWUE, (P_N_/gs) (**e**). Within each parameter, *, **, *** and **** represent significant differences for *p* ≤ 0.05, 0.01, 0.001 and 0.0001, respectively. Values are expressed as the mean and standard deviation (n = 6).

P_N_ was only significantly affected in UV-A 1 h and 4 h conditions (*p* < 0.01 and 0.0001, respectively) (Figure 2a), but Ci remained constant in all treatments (Figure 2b). Additionally, the exposure to UV-A 4 h and to both UV-B conditions significantly decreased (*p* < 0.0001 for UV-A 4 h and *p* < 0.05 for both UV-B) the gs (Figure 2c). Despite the similar E profiles in the different treatments, significant changes (*p* < 0.001) occurred at UV-A 4 h (Figure 2d). The intrinsic water use efficiency (iWUE), while showing a tendency to decrease with UV-A, was not significantly affected by any treatment (Figure 2e). 

**Figure 3:**
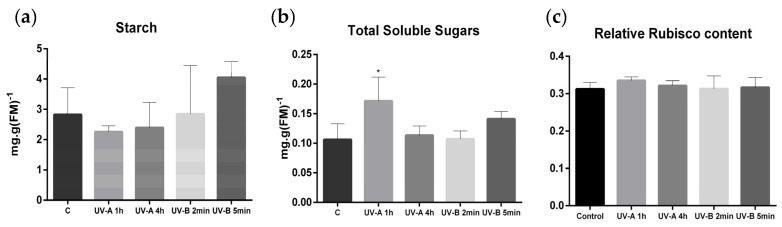
Carbon fixation in plants exposed 30 d to different UV-A/B conditions. Starch (**a**), Total Soluble Sugars (**b**), and Relative RuBisCO content (**c**) values. FM (Fresh Matter). Within each parameter, * represent significant differences for *p* < 0.05. Values are expressed as the mean and standard deviation (n = 10).

After 30 d of irradiation, the content of TSS significantly increased in the UV-A 1 h treatment (*p* < 0.05), whilst the starch levels showed a trend to decrease and increase with both UV-A and UV-B treatments, respectively (Figure 3a,b). The relative amount of RuBisCO was not affected by UV supplementation (Figure 3c).

### 2.4. Gene Expression for RuBisCO and PSII

**Figure 4:**
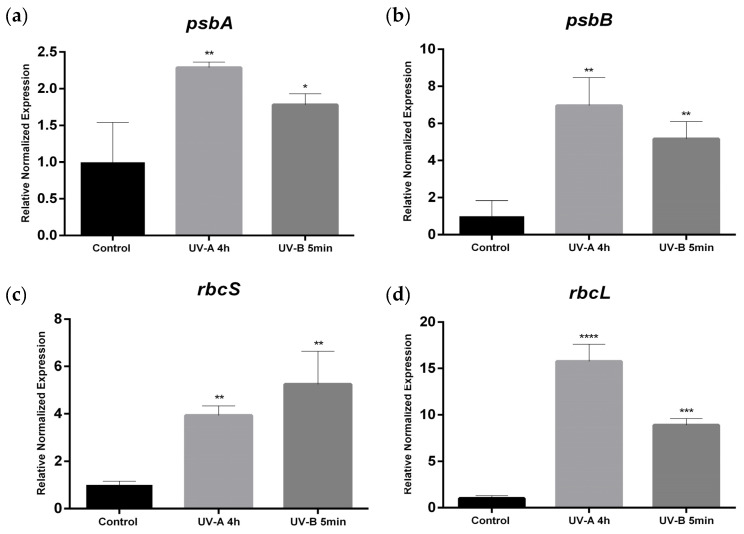
Regulation of photosynthesis pathway genes by different UV-A/B conditions. All parameters were measured in control, UV-A 4 h and UV-B 5 min (the last two are the higher exposure times for each radiation). The relative expression of the photosynthetic components was determined for psbA (**a**) and psbB (**b**) which encodes the D1 protein and CP47, respectively. At the same time, the relative expression of the genes encoding for the two subunits of RuBisCO were also assessed: rbcS (**c**) and rbcL (**d**) for small and large subunits, respectively. Within each parameter, *, **, *** and **** represent significant differences for *p* < 0.05, 0.01, 0.001 and 0.0001, respectively. Values are expressed as the mean and standard deviation. (n = 10).

The genes encoding for protein subunits of the PSII, D1 protein (psbA) and CP47 (psbB), showed a significant upregulation in all UV conditions, mostly UV-A 4 h (*p* < 0.01). UV-A 4 h and UV-B 5 min treatments induced a significant (*p* < 0.01 on rbcS and 0.0001, and 0.001, respectively, on rbcL) upregulation of the two genes encoding for the components of RuBisCO (large and small subunits) rbcS and rbcL. Except for rbcS, increases were higher on UV-A 4 h (Figure 4a–d).

### 2.5. Multivariate Approach

**Figure 5:**
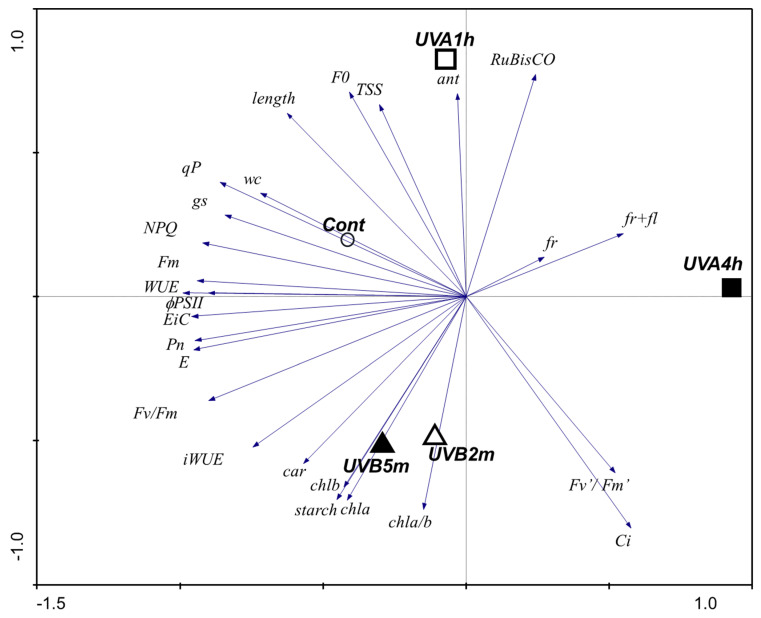
PCA analysis of functional responses of tomato fruit plants exposed to UV-A (1 and 4 h) and to UV-B (2 and 5 min) for 30 d.

Principal component analysis showed a clear separation between control and UV-B treatments (Figure 5). PC1 explained 43% of the variance and PC2 28% of the variance. Regarding the control, it is the most centered population being positioned on the top-left quadrant in the multivariance distribution. Both UV-B 2 min and UV-B 5 min scores are quite similar and are both located at the down-left quadrant, being mostly associated with higher levels of photosynthetic pigments, iWUE and starch. Conversely, the scores for UV-A 1 h and UV-A 4 h show that these two populations have different profiles, and both are highly different from the control and the UV-B scores. UV-A 1 h score is located at the the central-top and associated with increases in anthocyanins, F_0_, TSS, RuBisCO, while UV-A 4 h score is positioned at the right center and relates to fruits as well as fruits and flowers. 

## 3. Discussion

With the paradigm of producing “more with less”, the exponential population growth, and the alarming climate changes and pests and diseases that affect crops worldwide, the control of multiple variables in indoor production systems represent a new era of precision agriculture and a challenge to understand the mechanisms underlying the physiological and molecular responses of the crops to those variables. Mimicking natural conditions, including natural solar light, is a key challenge to increase the organoleptic and nutritional value of edible parts. Greenhouses create an ideal environment for intensive and precision crop production. However, they are usually built with UV-absorbing materials that reduce the benefits of UV-A and UV-B on crops along their life cycle. Comparing with their outdoor growing counterparts, crops growing indoors may have repressed metabolic pathways that are triggered by UV-A and UV-B plant sensors [32]. A previous study showed that the fruits produced indoors with the supplementation of low UV-A/B radiation had a higher quality to the consumers [11]; even so, it is necessary to ensure that this supplementation (quality and dose) is not too deleterious to plant growth and photosynthesis during fruiting and ripening stages. 

This work shows that UV-B may reduce plant length, principally with UV-B 2 min, which may indicate that at this low UV-B level, there is already an influence in cell division and cell expansion as reported for UV-B by Bandurska et al. [33]. Cell expansion depends on variables like the leaf water content, turgor pressure, and cell extensibility. This relation may thus support the correlation of length with %WC, shown by the PCA (Figure 5). This correlation also suggests an adjustment of the metabolism of the UV-treated tomato plants to restrict water use, which is supported by the reduced stomata aperture (Figure 5). 

The flowering and fruiting/ripening stages impose dramatic changes to the “source-sink” mobilization of photo-assimilates. In this process, leaf photosynthesis plays a critical role in the supply of carbohydrates to flowers/fruits. Under the stimulus of moderate UV-B, leaves shifted their metabolism to alternative secondary pathways, towards increasing, for example, flavonoids/phenols [34,35]. While this shift may enrich the edible parts with valuable secondary compounds increasing their nutraceutical and/or sensorial value [11], it also implies that the leaves may suffer a decrease in the necessary photo-assimilates to supply the fruits. 

As can be seen from the PCA, the low levels of UV-B and UV-A used here differently affect the photosynthetic parameters. UV-B 5 min increased Chl *a* and Chl *b*, supporting that UV-B stimulates the pathways involved in chlorophyll synthesis. Likewise, a short-term pre-harvest supplement of UV-B did not compromise the levels of Chl *a*, Chl *b*, or carotenoids in *Ocimum basilicum* leaves [26]. In tomato, the UV-B 5 min dose used stimulated the carotenoid pathways, supporting investment in these UV-protecting pigments and scavengers of reactive oxygen species (ROS), towards protecting chlorophylls from photo-oxidative damage caused by any excessive UV-B irradiation, as also proposed by Yadav et al. [29]. Phenolic compounds are also important protective compounds, associated with sensorial attributes (smell and taste). At these doses, UV-A has little impact on pigment levels, but compromises the efficiency of PSII photochemistry, although not significantly compromising the plant’s performance, growth, or fruit yield. Mariz-Ponte et al. [11] used the same doses of UV-A and demonstrated that, besides stimulating the antioxidant and antiradical activity of the fruit, and richness in phenolic compounds (e.g., flavonoids), UV-A also led to fruits more attractive to the consumers, and thus more likable to buyers. These beneficial aspects occur despite the negative correlation of UV-A with some photosynthetic parameters in the same plants, as shown here. The Fv/Fm ratio is a widely used indicator of photoinhibition or other injuries at the PSII complexes [36]. However, the UV-A plants were able to maintain Fv/Fm values close to 0.8, despite the decrease in UV-A conditions, showing a ratio characteristic of unstressed plants [37].

**Figure 6:**
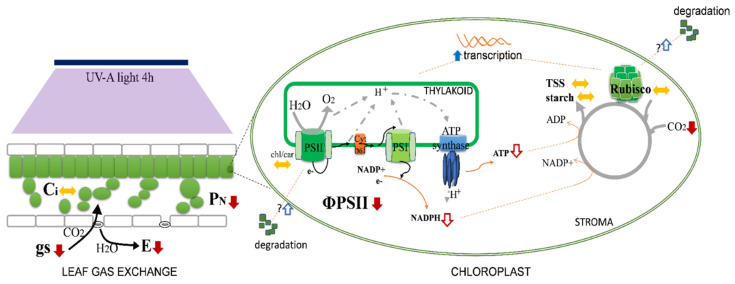
Major photosynthetic impacts and changes induced by moderate UV-A 4 h/day, for 30 d, in tomato flowering plants. Overall, the PSII fluorescence is affected by the decreased efficiency of ΦPSII, although LHC-pigment (Chl/car = chlorophyll/carotenoids) levels are not affected. This leads to fewer electrons being transported and thus a decrease in NADPH and ATP production and availability for the Calvin cycle. This reduction is related with the decrease in the net photosynthetic rate (P_N_), meaning that internal CO_2_ concentration (Ci) is not so depleted, and the stomatal conductance (gs) may decrease, therefore decreasing transpiration rate (E). Simultaneously, a degradation of RuBisCO may occur, but it can be replaced by new protein due to the stimulated accumulation of its transcripts (and increase its transcription), which overall may reset the negative impacts on the Calvin cycle, thus not having negative impacts on total amounts of soluble sugars and starch. Solid red arrows mean a decrease and solid blue arrows an increase. Dashed red and blue arrows mean a putative decrease and increase, respectively.

The profile shown by NPQ in response to UV-A (a stimulation for lower doses and a drastic decrease with the increase in the dose, comparing to the control) suggests a hormesis effect, meaning that lower doses of UV-A promote heat dissipation (which includes photo-protective mechanisms), while higher doses compromise this strategy. NPQ of chlorophyll *a* fluorescence is an indicator of the level of non-radiative energy dissipation in the light-harvesting complex of PSII (LHC II), which is attributed to the prevention of electron transfer chain over-reduction, preventing photodamage. The decrease in NPQ observed at UV-A 4 h may be supported by the decrease in the light-harvesting antenna size (lowered by Fm) and/or by other causes of PSII inactivation [36]. The reaction centers, which are functionally involved in the qP, are stimulated by the lowest dose of UV-A. Interestingly, the higher UV-A dose tested here decreased both the availability of the reaction centers to receive photons and ΦPSII, which suggests that UV-A 4 h already imposes some mild stress to plants (Figure 6). This mild stress may be the basis for the biochemical changes observed in the fruits as previously described [11]. Conversely, UV-B did not induce functional stress on the fluorescence/quenching parameters, but rather changes in the protective LHC-pigments composition, thus meaning a structural change.

We unveil for the first time the relationship between genes involved in RuBisCO response to high doses of UV-A/B light supplementation versus tomato plants growing without UVs. These data showed a tendency (*p* < 0.05) to increase the RuBisCO transcripts in the high doses of UV-A/B tested, contrary to that demonstrated on pea leaves under UV-B [38]. UV-A acts at the level of transcriptional regulation by increasing the transcripts of *psbB*, which encode for CP47. This protein is located at the antenna pigment complex CP43-47 and binds to chlorophylls and carotenes, acting in the transfer of energy from the LHC antenna to the photochemical reaction center. The transcript *psbA* encoding for the protein D1 (a protein involved in receiving electrons in the PSII) presented a similar behavior. D1 protein has shown susceptibility to UV-light [39,40]. Additionally, UV-A increased several proteins of the PSII in *Taxus* ssp. plants [41]. Based on these findings, we hypothesize that in UV-exposed tomato leaves, there might be a light-induced degradation of these proteins in the PSII, compensated by an increase in transcription to synthesize new proteins. Nevertheless, the possibility, within a process adaptive to UV radiation, that new PSII centers may be under generation to compensate the lower ΦPSII, should also be considered.

The PCA clearly shows that UV-A 4 h has an impact in the gas exchange parameters by the negative correlation of the vectors. While stomatal closure occurred and P_N_ decreased, the levels of Ci remained unchanged, which together with the increase in RuBisCO transcriptional levels may also support a higher investment on more RuBisCO protein to support Calvin cycle dynamics (Figure 6). This is supported by the increase in both *rbcL* and *rbcS* transcripts, which show that these high doses of UV already stimulated the synthesis of RuBisCO subunits. On the other hand, the fact that the relative content of RuBisCO is not affected supports the idea that UV might also induce RuBisCO degradation, which is offset by an increase in the transcript levels, allowing the maintenance of soluble sugars and/or starch in UV-supplemented leaves.

In conclusion, we have demonstrated previously that UV-A/B supplementation of tomato plants during fruiting/ripening increases the number of fruits with a richer nutraceutical composition and their preference by consumers [11]. Using the same UV-supplementation conditions described by Mariz-Ponte et al. [11], we demonstrate here that both UV light quality and dose differently target the leaf photosynthesis during the fruiting/ripening stage of the plant. UV-A had more positive and negative effects on photosynthetic functional parameters, while UV-B acted more at the structural level (stimulating pigment levels). The identification of targets specifically susceptible to UV quality/quantity light may provide a useful tool to selectively use UV-supplementation to produce better fruits (increased nutraceutical and organoleptic value) without detrimental effects on the photosynthesis of the plant, thus improving greenhouse crop production, with UV-A treatment for 1 h presenting potential as a better supplementation for fruit quality.

## 4. Materials and Methods

Plant material and experimental conditions: *Solanum lycopersicum* L. cv. ‘MicroTom’ seeds from JustSeed (UK) were germinated in the dark on individual pots containing 0.9 L of peat: perlite (2:1) substrate (n = 10/light treatment). Seedlings/plants were grown on the same pots under controlled conditions, at a photosynthetic photon flux density (PPFD) of 200 µmol m^−2^ s^−1^ of maximum light intensity (Fluorescent light by OSRAM L 30 W/77 FLUORA lamps), 23 ± 2 °C, 45 ± 5% relative humidity, and 16 h:8 h light photoperiod: dark. Seedlings/plants were watered with standard Hoagland medium with pH adjusted to 5.7 ± 0.05. After 90 d, plants started flowering, reaching maximum synchronization at day 100 after germination.

All light experiments were performed in a controlled growth chamber, following the recommended practices for UV described by Aphalo and Albert, [42]. For 30 d, starting on the 100th day of growing stage, five groups (n = 10) of flowering plants were supplemented daily with different UV radiation: Control Group—plants were maintained under the same irradiation conditions, with no UV supplementation; UV-A 1 h Group—plants were exposed for 1 h per day to a biologically effective irradiance (PG—plant growth spectrum) according to Flint and Caldwell, [43] of 0.00493 kJ m^−2^ UV-A supplied by blacklight lamps (F20T12/BLB—20 W T12 (T10) Fluorescent Blacklight Blue, with a peak wavelength at 368 nm); UV-A 4 h Group—plants were exposed for 4 h/day to 0.01972 kJ m^−2^ biologically effective irradiance of UV-A, supplied by the same UV-A lamps; UV-B 2 min Group—plants were exposed for 2 min/day to a biologically effective irradiance of 0.03057 kJ m^−2^ UV-B, supplied by six TFP-M/WL 8 W lamps with a peak wavelength at 312 nm; UV-B 5 min Group—plants were exposed for 5 min/day to 0.07642 kJ m^−2^ UV-B, supplied with the same UV-B lamps. UV lamps were placed 15 cm above the plants’ top. UV-A and UV-B light irradiance were measured, respectively, using the radiometer Phillip Haris (model: SEL240; Accrington, UK) and the radiometer International Light INC (model: IL1400; Newburyport, MA, USA). The energy irradiance ratio was calculated and multiplicated ×1000: UV-A: PAR (18.35) and UV-B: PAR (67.43). The biologically effective UV irradiance was calculated according to Flint and Caldwell, [43]: SpPG(λ) = exp [(4.688272 × exp (−exp (0.1703411 × (λ − 307.867)/1.15)) + ((390 − λ)/121.7557 − 4.183832))] if λ ≤ 390 nm; λ = wavelength in nm. For defining daily “low dose” of UV-B irradiation, we followed the range of 0.17–6.60 kJ·m^−2^ according to Escobar-Bravo et al. [13] and Soriano et al. [25] and for “low” daily exposure irradiances of UV-A we followed the range of 0.58–2.34 kJ m^−2^ [30].

Phenological data and plant growth: The total numbers of flowers and fruits, were determined using ten different plants (n = 10), after 30 days of exposure to the different treatments. Furthermore, shoot length, dry mass/fresh mass ratio (DM/FM), and water content percentage [%WC = ((FM − DM)/FM) × 100] were determined at the same time. Additionally, other morphological aspects (e.g., senescence, chlorosis, necrotic spots) were registered.

Chlorophyll *a* fluorescence and pigments: Chlorophyll *a* fluorescence was measured in fully expanded leaves at 12 *p*.m., resorting to the LI-COR 6400XT (LI-COR Biosciences, Nebraska, USA) using six plants per treatment. Dark-adapted leaves (30 min) were used to measure the minimal fluorescence yield (F_0_) after a weak modulated light, and the maximum fluorescence (Fm) after applying a saturating pulse of white light for 0.7 s. The variable fluorescence (Fv = Fm − F_0_) and the maximum quantum efficiency of PSII [Fv/Fm = (Fm − F_0_)/Fm] were calculated. Leaves were then adapted to light (30 min), and the steady-state fluorescence (Fs’), the maximal fluorescence (Fm’), and minimal fluorescence (F_0_’) were determined, after applying an actinic light, a saturating pulse and a weak modulate light, respectively, for 0.7 s. Additionally, the PSII maximum efficiency [Fv’/Fm’ = (Fm’ − F_0_’)/Fm’], the effective efficiency of PSII [ΦPSII = (Fm’ − Fs)/Fm’], photochemical quenching [qP = (Fm’ − Fs’)/(Fm’ − F_0_’)], and non-photochemical quenching [NPQ = (Fm − Fm’)/Fm’] were calculated according to Maxwell and Johnson [37] and Murchie and Lawson [44].

For the quantification of pigments, 10 leaves (from 3rd upper node) from different plants were collected in each treatment. Pigments were extracted with acetone:50 mM Tris buffer (80:20, *v*/*v*) pH 7.8 and centrifuged for 10 min at 10,000 g at 4 °C. Chlorophyll *a* (Chl *a*), chlorophyll *b* (Chl *b*), carotenoids (Car), and anthocyanins (Ant) contents were quantified by reading the absorbance at 470, 537, 647, and 663 nm in a multiplate reader Thermo Fisher Scientific Spectrophotometer (with three technical replicates per sample) [45].

Gas exchange measurements: Gas exchange was analyzed in six plants of each treatment using the LI-COR 6400XT (LI-COR Biosciences, Lincoln, NE, USA). Measurements took place at midday of the day, under atmospheric CO_2_ concentration and a saturating PPFD of 200 µmol m^−2^ s^−1^. Individual parameters were determined, including the transpiration rate (E, mmol (H_2_O) m^−2^ s^−1^), the stomatal conductance (gs, mmol (H_2_O) m^−2^ s^−1^), net photosynthetic rate (P_N_, µmol (CO_2_) m^−2^ s^−1^), and the intercellular CO_2_ concentration (Ci, ppm) [35]. Additionally, the intrinsic water-use efficiency (iWUE = PN/gs) (µmol (CO_2_) mol (H_2_O)^−1^) was calculated.

Carbohydrate content and RuBisCO relative quantification: Total soluble sugars (TSS) and starch contents were quantified through the anthrone method, using four replicates of leaf pools from 10 plants and quantified by a multiplate reader Multiskan Go (Thermo Fisher Scientific, Waltham, MA, USA) [16].

Leaf soluble proteins were extracted and quantified by the Bradford method (Sigma-Aldrich, St. Louis, MO, USA). For the quantification of the RuBisCO subunits, 15 µg of proteins were separated by SDS-PAGE, and gels were stained with 0.25% of Coomassie Brilliant Blue R250 as described by Li et al. [46]. Protein bands were compared with a protein molecular weight marker (SM0441, Fermentas, Vilnius, Lithuania). For the determination of the relative RuBisCO content, bands of the large and small subunits of each sample were isolated, overnight incubated in formamide (2 mL) at 50 °C, and the absorbance measured at 595 nm. The results are expressed as ABSRC/ABSTPC, in which RC is the RuBisCO content, and TPC is the total soluble protein content.

Transcriptional levels of genes related to the electron transport chain and RuBisCO: After assessing the biochemical results, only the highest doses of UV-A and UV-B were analyzed for transcriptional regulation. Total RNA of tomato leaves was isolated using PureZOL™ RNA Isolation protocol (Bio-Rad, Hercules, CA, USA), following the manufacturer’s instructions. For Reverse Transcriptase-PCR, RNA samples were treated with DNAse using Deoxyribonuclease I, Amplification Grade (Invitrogen™, Carlsbad, CA, USA). cDNA was obtained using NZY First-Strand cDNA Synthesis Kit, no oligos, NZYTech™ from 1 μg of RNA, following manufacturer’s instructions. Subsequently, cDNA was treated with 1 µL NZY RNase H, diluted with Milli-Q water and stored at −20 °C. Primers of two housekeeping genes [47,48] were used: the *elongation factor 1alpha* (*ef1*): 5′-TGGCCCTACTGGTTTGACAACTG-3′ (forward, f) and 5′-CACAGTTCACTTCCCCTTCTTCTG-3′ (reverse, r) and *ubiquitin* (*ubi*) gene: 5′-GGACGGACGTACTCTAGCTGAT-3′ (f) and 5′-AGCTTTCGACCTCAAGGGTA-3′ (r). For photosynthetic gene expression, we used genes coding for PSII proteins: D1 (*psbA*): 5´-TGGATGGTTTGGTGTTTTGATG-3′ (f) and 5′-CCGTAAAGTAGAGACCCTGAAAC-3′ (r); CP47 (*psbB*): 5′-CCTATTCCATCTTAGCGTCCG-3′ (f) and 5′-TTGCCGAACCATACCACATAG-3′ (r). Primers for the two genes encoding RuBisCO subunits were selected: large subunit (*rbcL*): 5′-ATCTTGCTCGGGAAGGTAATG-3′ (f) and 5′-TCTTTCCATACCTCACAAGCAG-3′ (r); and small subunit (*rbcS*): 5′-TGAGACTGAGCACGGATTTG-3′ (f) and 5′-TTTAGCCTCTTGAACCTCAGC-3′ (r). Real-time quantitative polymerase chain reaction (RT-qPCR) procedures were conducted in a CFX96 Touch™ thermocycler (Bio-Rad Laboratories, Hercules, CA, USA), using 2.5 µL of total first-strand cDNA, 5 µL of an enzyme (iTaq™ Universal SYBR^®^ Green Supermix, Bio-Rad), and 2.5 µL of primers (f + r), in a total volume of 10 µL. Amplifications were standardized with the conditions: 95 °C for 1 min followed by 40 cycles of 5 s at 95 °C and 30 s at 60 °C. The melting curve analysis ranged from 65 °C to 95 °C with increased temperatures of 0.5 °C in 5 s/cycle.

Statistical analysis: Except when mentioned otherwise, 10 plants were used in each condition, treated as individual samples, or treated as pools (pigments, carbohydrates, RuBisCO content determination and gene expression). At least three independent technical replicates were done for each experiment. Values are presented as mean ± standard deviation. Comparisons between the different treatments used the One-way ANOVA test (Graphpad™ Prism 6, San Diego, CA, USA). When data were statistically different, the Dunnett Comparison Test (*p* < 0.05) was also applied. For Multivariate analyses of data correlation, CANOCO for Windows v4.02 program was used for the Principal Component Analysis.

## Figures and Tables

**Table 1:** Growing and fruit production of *S. lycopersicum* plants exposed for thirty days to different UV-A/B conditions and control. These parameters were recorded in the last day of the experiment. Plant length (cm), leaf dry matter per unit of fresh matter (mg g(FM)^−1^), water content (%), number of flowers as well as flowers and fruits values. For the same parameter, * and ** represent statistically significant differences for *p* ≤ 0.05 and 0.01, respectively. Values are expressed as the mean and standard deviation (n = 10).

Treatment	Plant Length	WC	Leaf DM	Flowers	Flowers and Fruits ^a^
Control	20.6 ± 2.4	95.07 ± 2.62	0.139 ± 0.061	15.0 ± 6.3	30.7 ± 6.4
UV-A 1 h	19.4 ± 2.5	93.48 ± 0.95	0.204 ± 0.015 *	11.9 ± 5.2	41.6 ± 11.9 *
UV-A 4 h	16.4 ± 3.8	91.47 ± 0.95 **	0.185 ± 0.023	15.8 ± 7.0	43.2 ± 10.0 *
UV-B 2 min	16.6 ± 2.3 *	91.65 ± 0.83 **	0.194 ± 0.021	10.2 ± 4.8	41.0 ± 12.7
UV-B 5 min	17.7 ± 3.3	93.61 ± 1.10	0.216 ± 0.028 **	12.4 ± 6.7	34.7 ± 9.0

^a^: Number of fruits data retrieved from Mariz-Ponte et al. [11].

**Table 2:** Pigment contents in leaves from plants exposed for 30 d to different UV-A/B conditions and control. Chlorophyll *a* and *b* (Chl a and Chl b) (mg g^−1^ FM), chlorophyll ratio a/b (Chl a/Chl b), carotenoids (car) (mg g(FM)^−1^) and anthocyanins (ant) (µmol g(FM)^−1^) values. Within each parameter, *, **, and **** represent significant differences for *p* < 0.05, 0.01, and 0.0001, respectively. Values are expressed as the mean and standard deviation (n = 10).

Treatment	Chl *a*	Chl *b*	Chl *a*/*b*	Car	Ant
Control	1.44 ± 0.183	0.80 ± 0.073	1.79 ± 0.077	0.45 ± 0.030	0.050 ± 0.002
UV-A 1 h	1.45 ± 0.163	0.80 ± 0.088	1.81 ± 0.006	0.40 ± 0.041	0.046 ± 0.001 *
UV-A 4 h	1.40 ± 0.073	0.77 ± 0.030	1.82 ± 0.042	0.39 ± 0.014	0.045 ± 0.001 **
UV-B 2 min	1.69 ± 0.101	0.89 ± 0.059	1.90 ± 0.012 *	0.43 ± 0.031	0.040 ± 0.001 ****
UV-B 5 min	2.04 ± 0.088 **	1.10 ± 0.063 **	1.86 ± 0.027	0.54 ± 0.033 *	0.040 ± 0.001 ****

## Data Availability

To access these data, please contact the corresponding author.
